# Incidence, Clinical Characteristics and Outcomes of Severe Prosthesis–Patient Mismatch in Patients Undergoing TAVI with Large Aortic Annuli

**DOI:** 10.3390/medicina62050892

**Published:** 2026-05-06

**Authors:** Mohamed Ali, Muntaser Omari, Debbie Stewart, Sarah Lamb, Timothy Cartlidge, Rajiv Das, Richard Edwards, Azfar Zaman, Mohamed Farag, Mohammad Alkhalil

**Affiliations:** 1 Cardiothoracic Centre, Freeman Hospital, Newcastle-upon-Tyne NE7 7DN, UK; 2 Translational and Clinical Research Institute, Newcastle University, Newcastle-upon-Tyne NE1 7RU, UK

**Keywords:** aortic stenosis, TAVI, large annulus, prosthesis–patient mismatch, mortality

## Abstract

*Background and Objectives*: Recent studies have focused on evaluating the hemodynamic results in patients undergoing transcatheter aortic valve implantation (TAVI) with small aortic annuli. There is limited data on the incidence, clinical characteristics, and mortality of prosthesis–patient mismatch (PPM) in patients undergoing TAVI with large aortic annuli. *Materials and Methods*: This is a retrospective analysis of consecutive patients with severe aortic stenosis and large annuli who underwent TAVI at a single UK center. PPM was defined according to the Valve Academic Research Consortium (VARC-3) criteria and identified using echocardiography within 4–6 weeks following TAVI. Measurements were analyzed by an experienced operator who was blinded to the type of valve platform and clinical outcomes. *Results*: A total of 447 patients were screened, of whom 353 patients were included in the analysis. The incidence of any PPM or severe PPM was 38% and 15% of patients, respectively. Patients with severe PPM were younger, had larger body surface area, and were more likely to receive a balloon-expandable valve (BEV). At a mean follow-up of 35 months, mortality was numerically higher in patients with severe PPM (46% vs. 36%, *p* = 0.20) but this did not reach statistical significance. Similar mortality rates were observed among patients with or without severe PPM in those who received SEV as well as BEV. There was a differential role of body surface area in mortality in patients who developed severe PPM versus non-severe PPM. *Conclusions*: Severe PPM was evident in patients with large aortic annuli undergoing TAVI, particularly those who received BEV. Nonetheless, severe PPM did not impact mortality rate at three-year follow-up. Longer-term follow-up may be required to assess the impact of severe PPM on mortality.

## 1. Introduction

Transcatheter aortic valve implantation (TAVI) is now considered a treatment option for patients with severe aortic stenosis who are at low surgical risk [1,2,3]. More focus is placed on assessing long-term valve performance and durability given the expanded TAVI indication to include younger patients with severe aortic stenosis. Post-procedural hemodynamics and development of prosthesis–patient mismatch (PPM) are recognized to have an impact on long-term survival in patients undergoing aortic valve interventions [4,5].

In a large meta-analysis of 65 studies including more than 120,000 patients who underwent surgical aortic valve replacement, severe PPM was associated with 29% increased risk of death at long-term follow-up [4]. Similarly, in a meta-analysis assessing the outcome of PPM in patients undergoing TAVI, severe PPM was associated with 25% increased risk of death compared to no PPM [5]. Therefore, PPM is recognized as a non-structural type of bioprosthetic valve dysfunction according to the Valve Academic Research Consortium (VARC-3) criteria [6]. PPM is defined as an inappropriately small effective orifice area (iEOA) relative to body size, i.e., less than 0.85 cm^2^/m^2^. A recent update has used a lower cut point in obese patients of less than 0.70 cm^2^/m [6,7].

Recent studies have focused on characterizing PPM in patients with small aortic annuli undergoing aortic valve interventions, particularly TAVI [8,9,10,11]. In contrast, patients with large annuli have been studied less, with limited data on the incidence and the role of PPM in patients with severe aortic stenosis undergoing TAVI. Moreover, the impact of valve platforms in terms of using a self-expanding (SEV) versus balloon-expandable valve (BEV) on PPM and long-term outcomes remains to be determined. Therefore, the aim of this study was to evaluate the incidence, clinical characteristics, and long-term mortality associated with PPM in patients with large aortic annuli undergoing TAVI.

## 2. Materials and Methods

### 2.1. Study Design and Population

This was a retrospective, single-center observational study including consecutive patients with severe symptomatic aortic stenosis and large aortic annuli who underwent TAVI at the Freeman Hospital between March 2020 and 2024 (single UK center). Good Clinical Practice guidelines were applied during the study process and according to the Declaration of Helsinki. Anonymized data, including clinical and echocardiographic data, were obtained from the institutional database normally utilized for patient clinical care. Due to the retrospective nature of this study of anonymized data, informed consent was waived.

Severe aortic stenosis was defined according to the current guidelines using measured aortic valve area on transthoracic echocardiography criteria: an aortic valve area (AVA) of <1 cm^2^, an indexed AVA of <0.6 cm^2^/m^2^, a maximum transvalvular velocity of more than 4 m/s, or a mean transvalvular gradient of more than 40 mmHg. Patients with low-flow aortic stenosis who, following heart team discussion, fulfilled criteria for the diagnosis of severe aortic stenosis were also included in this study [1].

All patients underwent computed tomography (CT) as part of their work-up and for TAVI procedural planning. Only patients with large annuli were included in this analysis. Large annuli were defined using 3mensio software (version 10.7.3 3mensio Structural Heart, 3mensio Medical Imaging, Maastricht, The Netherlands), and an aortic annulus was considered large if the aortic annulus area was more than 440 mm^2^. This cut-off was derived from previous studies that assessed the role of annular size in patients undergoing TAVI procedures [8,9,11]. Patients with extra-large annuli were treated according to operator discretion using BEV or SEV.

### 2.2. TAVI Procedure

All procedures were performed by two experienced operators after discussion among the multidisciplinary heart team and using standard TAVI techniques. Valve type and size were selected based on pre-procedural computed tomography and using commercially available sizing charts. Balloon-expandable and self-expanding valve platforms were used according to operator discretion and anatomical considerations.

### 2.3. Echocardiographic Assessment and Definition of PPM

Transthoracic echocardiography was performed within four to six weeks following TAVI. The continuity equation was applied to calculate the effective orifice area and subsequently indexed to body surface area to identify any PPM. Left ventricular outflow tract (LVOT) diameter was measured as previously described [11,12]. In summary, this was done using the long-axis view during mid-systole and the LVOT diameter was measured from the leading edge to the leading edge just below the level of the aortic annulus. Pulsed-wave Doppler was used to measure the LVOT velocity-time integral (VTI), which was obtained in the apical 5-chamber view. Careful consideration was given to measure LVOT diameter and LVOT VTI from the same location. These measurements were performed ‘offline’ and were recorded by an experienced and accredited sonographer who was not aware of the aim of the study. All echocardiogram studies followed an internal protocol that is compliant with current guidelines on performing echocardiogram post TAVI. In cases wherein LVOT measurements were not reliable, LVOT diameter was obtained from echocardiograms pre-TAVI.

PPM was defined according to VARC-3 criteria as follows:

No PPM: indexed effective orifice area (iEOA) > 0.85 cm^2^/m^2^; moderate PPM: iEOA 0.66–0.85 cm^2^/m^2^; and severe PPM: iEOA ≤ 0.65 cm^2^/m^2^. A lower cut-off was used for patients with body mass index (BMI) of more than 30 kg/m^2^, as recommended by the VARC-3 criteria [6]. No PPM: indexed effective orifice area (iEOA) > 0.70 cm^2^/m^2^; moderate PPM: iEOA 0.56–0.70 cm^2^/m^2^; and severe PPM: iEOA ≤ 0.55 cm^2^/m^2^.

The primary outcome was all-cause mortality at the last available encounter. Mortality data were obtained from hospital records and national databases.

### 2.4. Statistical Analysis

Data was assessed for normality using the Shapiro–Wilk test. Continuous variables were compared using Student’s t-test or the Mann–Whitney U test, as appropriate, and were presented as the mean ± standard deviation or median with interquartile range according to their normality testing. Categorical variables were presented as counts and percentages and were compared using the chi-square test or Fisher’s exact test, as appropriate. To investigate the relationship between mortality and severe PPM, univariate and multivariable hazard regression models of Cox were used. All study variables were first analyzed with univariate analysis, and those that showed a significant interaction were entered into the final multivariable analysis, including variables that were considered clinically relevant such as age, gender and valve type. Survival analyses were performed using Kaplan–Meier estimates and compared with the log-rank test, and missing data were not imputed in the analysis. Statistical analyses were performed using SPSS 30.0 (SPSS, Inc., Chicago, IL, USA), and a *p* value < 0.05 was considered statistically significant.

## 3. Results

**Figure 1:**
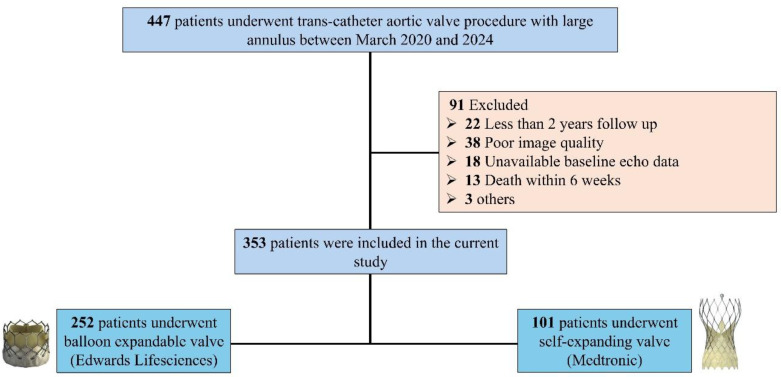
Study flow chart.

A total of 447 patients who underwent TAVI with large aortic annuli were screened. Patients who had less than two years of follow-up (4.9%), poor image quality (8.5%), or unavailable baseline echo (4.0%) or died within 6 weeks from the date of their procedure (2.9%) (of which eight cases were in-hospital, including three procedural deaths) were excluded from the analysis (Figure 1).

The mean age of the 353 included patients was 81 ± 7 years, and they were predominately males (76%). The average body surface area (BSA) was 1.9 ± 0.3 m^2^. The average valve area was 0.73 ± 0.21 cm^2^ with a mean gradient of 45 ± 15 mmHg and left ventricle ejection fraction of 50 ± 10%. Most patients underwent TAVI with the BEV platform (72%).

Based on VARC-3 criteria, the proportion of patients who had any degree of PPM or severe PPM was 38% and 15%, respectively. Patients with severe PPM were younger (79 ± 9 vs. 81 ± 7, *p* = 0.031) and had larger body surface area (BSA) (2.0 ± 0.2 vs. 1.9 ± 0.3, *p* = 0.03) compared to those without severe PPM. The remaining baseline clinical characteristics were comparable between the two groups, including the proportion of males (75% vs. 76%, *p* = 0.80) (Table 1).

There were no differences in the echocardiographic features among patients with or without severe PPM (Table 2). Notably, patients with severe PPM were more likely to receive the BEV platform (84% vs. 69%, *p* = 0.029) and a smaller-sized valve (26 mm: 78% vs. 48%, 29 mm: 18% vs. 40%, 34 mm: 4% vs. 10%, *p* < 0.001).

**Figure 2:**
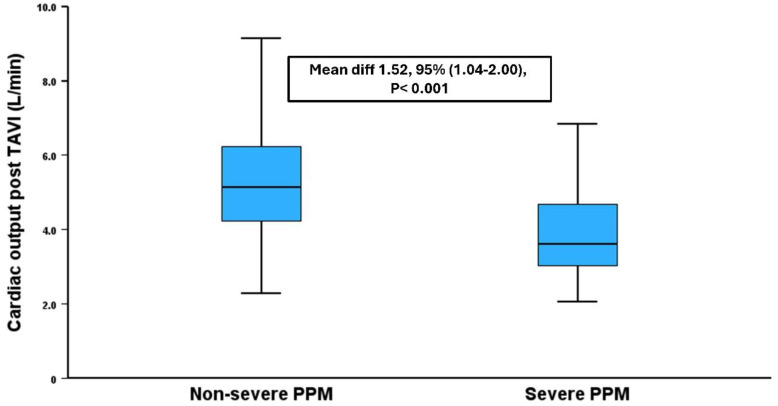
Difference in cardiac output between patients with large annuli according to their prosthesis–patient mismatch status.

Following the TAVI procedure, the suboptimal hemodynamic results were consistent in patients with severe PPM compared to those without severe PPM. This includes stroke volume (51 ± 13 vs. 76 ± 21 mL, *p* < 0.001), cardiac output (3.9 vs. 5.4 L/min, *p* < 0.001), and cardiac index (2.0 vs. 2.9 L/min/m^2^, *p* < 0.001) (Figure 2).

**Figure 3:**
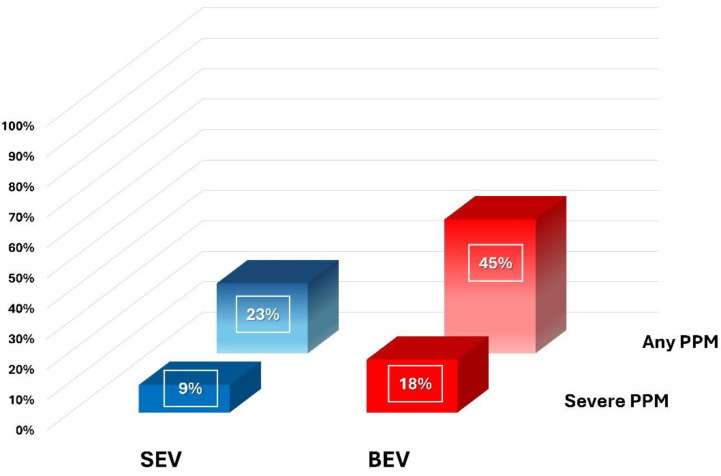
The incidence of any or severe prosthesis–patient mismatch according to the transcatheter heart valve platform. BEV: balloon-expandable valve, SEV: self-expandable valve.

Patients with the BEV platform were more likely to develop any degree of PPM (23% vs. 45%, *p* < 0.001) or severe PPM (9% vs. 18%, *p* = 0.029) compared to patients who received the SEV one (Figure 3).

**Figure 4:**
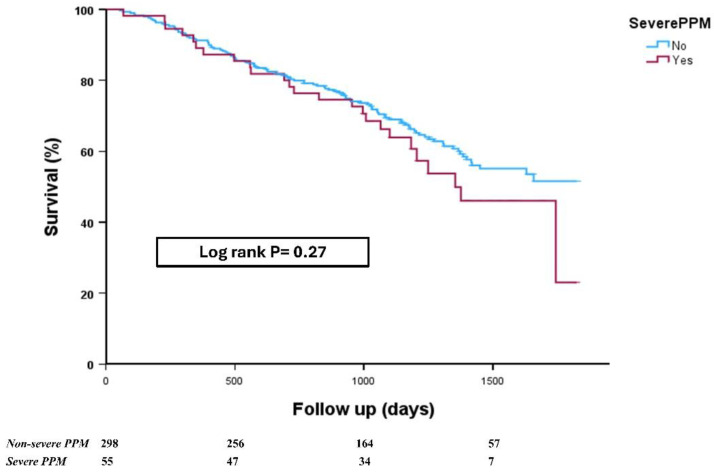
The primary endpoint of all-cause mortality in patients undergoing transcatheter aortic valve implantation according to their prosthesis–patient mismatch status. Kaplan–Meier curve of the cumulative incidence of all-cause death at mean follow-up of 35 months.

At a mean follow-up of 35 ± 15 months (almost 50% of patients completed 3 years of follow-up), there was a numerical difference in mortality among patients who developed severe PPM versus non-severe PPM (46% vs. 36%, log-rank *p* = 0.27), although this difference did not reach statistical significance (Figure 4). Comparable mortality was also reported even after adjustment to other variables such as age, gender, and valve type (hazard ratio HR 1.24, 95% confidence interval 0.78- 1.97, *p* = 0.37). This lack of difference was also evident in patients who underwent TAVI with the BEV (46% vs. 40%, *p* = 0.47) as well as SEV platform (44% vs. 28%, *p* = 0.31).

When the data was analyzed according to BSA, there was a difference in mortality in patients with small BSA (defined as less 2.0 m^2^) who developed severe PPM versus non-severe PPM (57% vs. 33%, *p* = 0.03). The mortality rate was comparable in patients with large BSA between the two groups (38% vs. 40%, *p* = 0.83). Notably, severe PPM was more frequently present in patients with small BSA undergoing BEV compared to SEV (14% vs. 3.7%, *p* = 0.042), but there was a similar rate in patients with large BSA (23.3% vs. 14.9%, *p* = 0.23).

## 4. Discussion

The main findings from this study can be summarized as follows: Firstly, PPM was present in patients with large aortic annuli and in its severe form can affect 15% of patients undergoing TAVI. Secondly, patients with PPM were younger, had larger BSA, and were more likely to receive the BEV platform. Thirdly, severe PPM did not impact mortality at three-year follow-up. Fourthly, body surface area, and not valve platform, may play a differential role in mid-term mortality in patients with large annuli undergoing TAVI.

Patients who are considered to be at low surgical risk are increasingly treated with TAVI [1]. Recent data suggest that TAVI is non-inferior and can be superior to surgical aortic valve replacement at one-year follow-up [13,14]. However, targeting younger populations requires careful evaluation of the long-term outcomes of transcatheter heart valve (THV) procedures, including the risk of developing PPM. Surgical as well as TAVI data have highlighted the association between PPM and long-term mortality [4,5]. However, studies have focused on patients with small aortic annuli, given the increased risk of PPM in this particular group [8,9,11]. In fact, recent studies have highlighted an increase in early mortality in patients who underwent TAVI with very small annuli and developed severe PPM [15].

Our study highlighted the prevalence of PPM in patients with large aortic annuli undergoing TAVI procedures. The presence of PPM was a relatively common finding, affecting more than one-third of patients. Notably, severe PPM, as defined by the VARC-3 criteria, was present in almost one in seven patients with large aortic annuli. This group of patients was younger, had larger body surface area, and was more likely to receive the BEV platform. These findings challenge the assumption that large annular dimensions protect against PPM. While larger annuli allow implantation of larger prostheses, patient body size and valve design appear to play an important role in determining post-procedural hemodynamics. Importantly, the BEV platform that was used in our study was SAPIEN 3 Ultra. This fourth generation of Edwards THV is recognized to have a restricted leaflet motion, which impacts the hemodynamic results post TAVI [16]. Recent development of SAPIEN Ultra Resilia allowed for better leaflet motion by redesigning their suspension at the commissures to optimize hemodynamic and clinical results [16,17]. This resulted in lower gradients and larger effective orifice area, although these changes were only applied to the 20 mm and 23 mm valve sizes. Whether similar results will be identified in the Resilia fifth generation, or other manufacturers, is yet to be determined.

Despite the observed differences in PPM incidence, we did not find a statistically significant association between PPM and mortality. This may reflect limited statistical power, the relatively small number of patients with severe PPM, or the relatively short-term follow-up of almost three years. Both surgical and transcatheter data highlighted that mortality rates diverge after 3-year follow-up [4,5]. Therefore, longer-term follow-up may yield different results, which can be supported by the significant increase in mortality towards the end of the study period in patients who developed PPM. Importantly, mortality outcomes were similar across valve platforms, suggesting that valve selection should be individualized based on anatomical and procedural considerations. Both SEV and BEV can safely be used to treat severe aortic stenosis patients with large aortic annuli [18,19].

Our results highlighted the significant difference in cardiac output, cardiac index and stroke volume according to the PPM status. Patients who developed PPM had worse hemodynamic results following the TAVI procedure. This difference may result in worse symptomatic status and may lead to more frequent heart failure admissions. Previous studies have demonstrated the increased risk of heart failure admissions in patients who develop PPM [4,20]. Moreover, PPM may affect functional capacity, resulting in poor quality of life, although data is not consistent [21,22]. Our data support this finding and provide mechanistic insights as to why patients with PPM reported worse health status compared to those without PPM. Future adequately sized studies need to address this question, and this remains a hypothesis-generating concept.

The current analysis illustrated that BSA has a differential impact on mortality in patients with PPM. Patients with small BSA who developed PPM were more likely to sustain mortality events than those without PPM. This group of patients is not expected to develop PPM, given their small BSA. Therefore, it is plausible that the presence of PPM may not solely be related to the mismatch between body size and effective valve area but potentially reflect an intrinsic issue with the implanted THV. This may include malpositioning, recoil, or significant underexpansion that can restrict leaflet opening, resulting in a small indexed effective orifice area. Therefore, recognition of PPM in patients with large annuli but small BSA is important following TAVI. Detailed imaging using CT may add further insights into the mechanisms behind its development and may provide a rationale to subject patients to further post-dilatation to reduce risk of PPM. Additionally, patients with small BSA who developed severe PPM were more likely to have received BEV. Whilst the design of the valve may play a role here, the observational nature of the study would preclude any definitive conclusions, and future randomized studies are needed to address this question. Additionally, this analysis is considered hypothesis-generating and dedicated studies are required to address this research question, particularly using the current BSA cut-off.

### Limitations

This study has several limitations, including its retrospective design and single-center setting. Residual confounding cannot be excluded. Echocardiographic assessment was performed at a single time point after TAVI, and changes in valve hemodynamics over time were not evaluated. Finally, although follow-up was relatively short, and the sample size may have limited ability in detecting small differences in mortality.

## 5. Conclusions

PPM was evident in patients with large aortic annuli undergoing TAVI, particularly those who received BEV. Nonetheless, PPM did not impact mortality rate at three-year follow-up. Longer-term follow-up may be required to assess the impact of PPM on mortality.

## Figures and Tables

**Table 1:** Baseline clinical characteristics of patients with large annuli, stratified according to the presence of severe prosthesis–patient mismatch (PPM).

	Whole Cohort n = 353	Non-Severe PPM n = 298	Severe PPM n = 55	*p* Value
Age (mean ± SD)	81 ± 7	81 ± 7	79 ± 9	0.031
Male gender (n, %)	268 (76%)	227 (76%)	41 (75%)	0.80
Body surface area (mean ± SD)	1.9 ± 0.3	1.9 ± 0.3	2.0 ± 0.2	0.03
Body mass index	28 ± 6	28 ± 6	28 ± 7	0.36
NYHA III/IV (n, %)	275 (78%)	232 (78%)	43 (78%)	0.96
Elective admission (n, %)	257 (73%)	217 (73%)	40 (73%)	0.99
Diabetes (n, %)	87 (25%)	72 (24%)	15 (27%)	0.81
Smoker (n, %)	12 (3%)	9 (3%)	3 (6%)	0.36
Creatinine (mean ± SD)	113 ± 72	111 ± 75	118 ± 51	0.56
Chronic lung disease (n, %)	69 (20%)	57 (19%)	12 (22%)	0.64
Previous CVA/TIA (n, %)	43 (12%)	36 (12%)	7 (13%)	0.89
Atrial fibrillation (n, %)	98 (28%)	81 (28%)	17 (31%)	0.58
Previous pacemaker (n, %)	16 (5%)	14 (5%)	2 (4%)	0.73

CVA: cerebrovascular event; NYHA: New York Heart Association; TIA: transient ischemic attack.

**Table 2:** Echocardiographic and procedural characteristics of patients with large annuli, stratified according to the presence of severe prosthesis–patient mismatch (PPM).

	Whole Cohort n = 353	Non-Severe PPM n = 298	Severe PPM n = 55	*p* Value
Peak gradient (mean ± SD)	73 ± 24	74 ± 24	71 ± 23	0.54
Mean gradient (mean ± SD)	45 ± 15	45 ± 15	44 ± 16	0.76
Valve area (mean ± SD)	0.73 ± 0.21	0.74 ± 0.22	0.70 ± 0.18	0.32
LV function% (mean ± SD)	50 ± 10	50 ± 11	48 ± 9	0.25
Annulus area (mean ± SD)	509 ± 66	506 ± 66	537 ± 63	0.20
Predilatation (n, %)	96 (27%)	85 (29%)	11 (20%)	0.18
BEV (n, %)	252 (71%)	206 (69%)	46 (84%)	0.029
Valve sizes				<0.001
26 mm	189 (53%)	143 (48%)	43 (78%)	
29 mm	130 (37%)	120 (40%)	10 (18%)	
34 mm	32 (9%)	30 (10%)	2 (4%)	

BEV: balloon-expandable valve; LV: left ventricle.

## Data Availability

The data presented in this study are available on request from the corresponding author.
